# Draft Genome Sequence of a *Klebsiella pneumoniae* Strain (New Sequence Type 2357) Carrying Tn*3926*

**DOI:** 10.1128/genomeA.00986-16

**Published:** 2016-09-22

**Authors:** Xing-bei Weng, Zu-huang Mi, Chun-xin Wang, Jian-ming Zhu

**Affiliations:** a Department of Medical Laboratory, Ningbo First Hospital, Ningbo, China; b Department of Bioinformation, Wuxi Clon-Gen Technology Institute, Wuxi, China; c Department of Medical Laboratory, Wuxi People’s Hospital Affiliated to Nanjing Medical University, Wuxi, China; d Department of Medical Laboratory, Hangzhou Yuhang Hospital of Traditional Chinese Medicine, Hangzhou, China

## Abstract

We present the draft genome sequence of a *Klebsiella pneumoniae* carbapenemase–producing sequence type 2357 (ST2357) strain, NB60, which contains drug-resistant genes encoding resistance to beta-lactams, fluoroquinolones, aminoglycosides, trimethoprim-sulfamethoxazole, colistin, macrolides, and tetracycline. Strain NB60 was isolated from human blood, making it an important tool for studying *K. pneumoniae* pathogenesis.

## GENOME ANNOUNCEMENT

*Klebsiella pneumoniae* carbapenemase (KPC)–producing *Enterobacteriaceae* have spread worldwide and pose a significant threat in nosocomial infections, such as septicemia, pneumonia, and urinary tract infections (1). *K. pneumoniae* strain NB60 was isolated from the blood of a patient in Ningbo First Hospital, People’s Republic of China, in 2012. The strain belongs to the new multilocus sequence type 2357 (ST2357). The variety of virulence genes and resistant genes in the genome sequence of NB60 will thus serve as a useful resource for future studies into bacterial survival and antibiotic resistance of this important human pathogen.

NB60 was resistant to multiple antibiotics used clinically, including beta-lactams, fluoroquinolones, aminoglycosides, trimethoprim-sulfamethoxazole, colistin, macrolides, and tetracycline.

Next-generation sequencing was performed using an Illumina MiSeq instrument with 150-bp paired-end reads and ~500-fold coverage. *De novo* assembly was performed with Edena version 3 (2), ABySS version 1.3.1 (3), and Velvet version 1.2 (4). Reads were also aligned to *Klebsiella pneumoniae* NTUH_K2044 (GenBank accession no. NC_012731). The *de novo* assemblies and alignment-based contigs were merged using Gap4 (5) and scaffolded with SSPACE version 2.0 (6), and gaps were PCR-amplified and sequenced by Sanger sequencing. The assembly of the pseudochromosome resulted in 42 contigs, and 15 unmapped contigs, which were annotated via the NCBI Prokaryotic Genome Annotation Pipeline (PGAAP) (7). The pseudochromosome is 5.07 Mb and its G+C content is 57.43%. The unmapped contigs comprise 0.73 Mb with a G+C content of 48.39%. NB8 provides a total of 6,197 genes, 9 rRNAs (5S, 16S, 23S), and 78 tRNAs.

*In silico* genome analysis identified 17 antimicrobial resistance genes encoding resistance to beta-lactams (*bla*_KPC-2_, *bla*_CTX-M-14_, *bla*_CTX-M-15_, *bla*_OXA-1_, *bla*_SHV-2_), aminoglycosides [*ant*(3′’)-Ia, *aac*(6′)-Ib, *aph*(3′)-II, ksga], fluoroquinolones (*qnr*S1), macrolides (*mphA*), sulfonamide-trimethoprim (*sul*1, *dfrA12*), colistin (*arn*A), and tetracycline (*tet*A, 2 copies of *tet*C).

In addition, NB60 carried a Tn*21*-like transposon, Tn*3926*, which was also isolated from a strain of *K. pneumoniae* in Taiwan (8). And the genome encodes numerous transposases and insertion sequences, highlighting the importance of genomic exchange in creating the NB60 pathogenic phenotype.

The draft genome sequence of *K. pneumoniae* strain NB60 will aid in precise genetic manipulation and thereby further improve the study of KPC-producing *K. pneumoniae* virulence.

### Accession number(s).

The draft genome sequence of *Klebsiella pneumoniae* strain NB60 has been included in the GenBank whole-genome shotgun database under the accession number AZAP00000000.
